# Development of the InSynQ checklist: A tool for planning and reporting the synthesis questions in systematic reviews of interventions

**DOI:** 10.1002/cesm.12036

**Published:** 2023-12-12

**Authors:** Miranda S. Cumpston, Joanne E. McKenzie, Rebecca Ryan, Ella Flemyng, James Thomas, Sue E. Brennan

**Affiliations:** ^1^ Methods in Evidence Synthesis Unit, School of Public Health and Preventive Medicine Monash University Melbourne Victoria Australia; ^2^ Centre for Health Communication and Participation La Trobe University Melbourne Victoria Australia; ^3^ Cochrane London UK; ^4^ EPPI‐Centre, UCL Social Research Institute University College London London UK; ^5^ School of Public Health and Preventive Medicine Monash University Melbourne Victoria Australia

**Keywords:** evidence synthesis, PICO, reporting, reporting guideline, research questions, synthesis questions, systematic reviews

## Abstract

**Introduction:**

Methods guidance and appraisal tools for systematic reviews require specification of the question and eligibility criteria for the review (“PICO for the review”). Less emphasis has been given to specifying the question and criteria for each synthesis (“PICO for each synthesis”), yet decisions about which studies to include in each synthesis can critically influence the utility and findings of a review. This paper describes the rationale and methods for developing the InSynQ (Intervention Synthesis Questions) tool for planning and reporting synthesis questions in reviews of interventions. The aim is to provide transparency about the basis of the tool and contribute to evidence on methods for developing guidance for research conduct and reporting.

**Methods:**

Informed by EQUATOR Network methods, we (1) established a project group; (2) examined reporting of the “PICO for each synthesis” in published reviews; (3) reviewed existing reporting guidance and the *Cochrane Handbook for Systematic Reviews of Interventions*; (4) drafted items with elaboration, explanation, and examples; (5) conducted consultation meetings, an evaluation survey and pilot test; (6) incorporated feedback.

**Results:**

Existing reporting guidelines do not distinguish the review and synthesis PICO, with limited coverage of the elements needed to specify the PICO for each synthesis. Using the PRISMA 2020 format, the draft tool contained 10 items with essential and additional reporting elements, explanations, and examples. Revisions arising from consultation meetings (>30 people), included adding an eleventh item on consumer and stakeholder involvement, a figure explaining PICO for each synthesis, and integrating examples into elements/explanations. All respondents to the survey (12 people) said the tool would help them plan or appraise synthesis questions. InSynQ is available at https://InSynQ.info.

**Conclusion:**

Transparent reporting of the development process contributes to the evidence base for methods to develop guidance. It may improve uptake of InSynQ, in turn enhancing the clarity of syntheses.

## 1 BACKGROUND

Systematic reviews are a method of research used to identify, collate, appraise, and synthesise the available evidence to answer specific research questions. In doing so, they provide an evidence base to underpin decision making by health professionals, policy makers, the public and other researchers [1, 2]. An individual systematic review may address multiple research questions [3], for example, estimating the effects of different types of interventions for a health condition (such as whether changes to visiting rules, increasing physical distancing, or mask‐wearing are effective for reducing transmission of COVID‐19 in residential care settings [4]), or the same intervention among different types of people (such as whether the effects of rotavirus vaccines differ in countries with different underlying mortality rates) [5]. Careful planning of these questions can ensure more useful reviews focused on questions of importance to decision‐makers. It may also reduce bias in the review process, lessening the risk that decisions made when conducting the synthesis are driven by what is reported in the included studies. In turn, complete and unambiguous reporting of synthesis questions is crucial to decision‐makers' understanding of and trust in a review.

Addressing multiple questions within a review of intervention effects requires review authors to identify the subset of primary studies that address each synthesis question by examining the study PICO [6] (population(s), intervention(s), comparator(s), outcome(s)). In many ways, this step is analogous to the process of selecting studies for inclusion in a review, except that decisions are made at the level of the synthesis. Taking the COVID‐19 example above, authors will need to examine the intervention evaluated in each included study and determine which intervention group each study belongs in (“changes to visiting rules,” “physical distancing,” and so on). By defining each intervention group, review authors provide the criteria used to decide which studies are eligible for each synthesis. Just as decisions about which studies are eligible for a review can critically influence the findings and conclusions of the review, so too can decisions about eligibility for each synthesis. Specifying the eligibility criteria for each synthesis (i.e., the “PICO for synthesis”) enables authors to allocate the studies included in the review to each synthesis in a transparent and more reproducible manner (see Box 1 for examples). When done in advance, it should help reduce post hoc decision making about comparisons. Clear reporting should also increase clarity for readers in understanding how and why studies were grouped in a particular way.

Box 1 **Examples of using clearly defined and replicable groups to specify synthesis questions**

**Table:** 

**Population groups** In a review of vaccines for preventing rotavirus diarrhoea, the authors asked: Do rotavirus vaccines prevent severe rotavirus diarrhoea and deaths in children under five in countries with low child mortality rates, medium child mortality rates, and high child mortality rates? To address these questions, they provided a clear and replicable definition of the three population groups based on data from a UNICEF report on levels and trends in childhood mortality [7]: 1. Low‐mortality countries: those in the lowest quartile of under‐five child mortality rates 2. Medium‐mortality countries: those in the second quartile of under‐five child mortality rates 3. High‐mortality countries: those in the highest two quartiles of under‐five child mortality rates. The meta‐analyses of primary outcomes were then stratified by these three population groups, providing separate effect estimates for each group to answer each question [5].*Intervention groups In a review of nonpharmacological interventions for preventing COVID‐19 infections in long‐term care facilities, the authors asked: What are the effects of the different types of interventions that are used for preventing SARS‐CoV‐2 infection and spread? The authors structured the reporting of results according to four broad categories of intervention (points 1–4). Syntheses of effects were reported for each of the intervention groups listed in the bracketed text. A clear and replicable definition was provided for each group (not shown). 1. Entry regulations (e.g., full or partial closure to visitors, measures to reduce viral introduction through staff or new/returning residents) 2. Contact‐regulation and transmission‐reduction (e.g., cohorting within the facility, use of protective equipment) 3. Surveillance (e.g., testing and screening of staff and residents) 4. Outbreak control (e.g., symptom‐based targeted testing, contact tracing, and testing) [4]*

*Note: Original text has been edited to illustrate concepts.

While the reasons and process for specifying the PICO for each synthesis are analogous to those pertaining to the review as a whole, most methodological expectations for systematic reviews have not covered this aspect of planning and reporting [8, 9, 10] or have done so to a limited extent [11, 12, 13, 14, 15]. The aim of this paper is to describe the development of the InSynQ (Intervention Synthesis Questions) tool for planning and reporting the questions addressed in systematic reviews of interventions. The tool is intended for use in reviews of any type of intervention (health and other). The complete InSynQ checklist and guide are available at https://InSynQ.info.

## 2 METHODS

### 2.1 Context for this project

InSynQ is based on guidance from Chapters 2 and 3 in version 6 of the *Cochrane Handbook for Systematic Reviews of Interventions* (hereafter, the “Cochrane Handbook”) [3, 6, 16]. These chapters provide guidance for planning and defining the comparisons in a systematic review, and introduced the concept of the “PICO for each synthesis.” Following publication of the Cochrane Handbook chapters, we examined the reporting of the PICO for each synthesis in published reviews [17], with the aim of identifying common gaps in practice and the need for strategies to address these gaps.

A concurrent call from Cochrane [18] for proposals to develop practical tools to assist authors of systematic reviews to implement guidance from the Cochrane Handbook provided funding and the impetus to develop InSynQ. Cochrane's call focused on reviews of public health and health systems interventions. Interventions in these fields frequently feature diverse populations, diverse settings, and intervention complexity [19], requiring careful attention when defining research questions and planning the structure of the synthesis. While the examples used to illustrate reporting in the first version of InSynQ come from reviews of public health and health systems interventions, the items and content were written for systematic reviews examining the effects of any health intervention, and may also be applicable to reviews of interventions outside the field of health.

**Figure 1:**
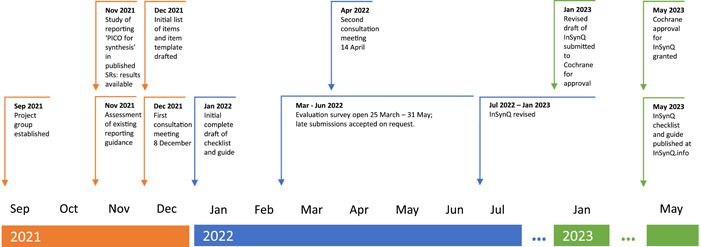
Timeline of development process for InSynQ checklist and guide.

The methods for this project were informed by guidance proposed by members of the EQUATOR Network for the development of reporting guidance [20]. To develop the InSynQ checklist and guide, we: (1) established a project group; (2) assessed existing practice by conducting a study of the reporting of the “PICO for each synthesis” in published systematic reviews; (3) identified possible content and established the need for new guidance by assessing the coverage and content of existing reporting guidance; (4) developed an initial draft checklist and guide, (5) convened online consultation meetings to gather feedback on the draft, (6) conducted an open evaluation survey to gather further feedback, and (7) revised the draft in light of the feedback received (see Figure 1).

### 2.2 Establishing the project group

The project group was established to commence work on InSynQ in September 2021, comprising the core team responsible for developing and drafting the guidance and checklist (SB and JM, principal investigators on the funding proposal; MC and RR investigators), along with a methodological advisor (JT, Senior Scientific Editor of the Cochrane Handbook) and an advisor responsible for coordinating the implementation of methods in Cochrane reviews (EF, Cochrane Evidence Production and Methods Directorate). The core team had experience designing and authoring Cochrane and other systematic reviews, editorial roles within Cochrane (MC, JM, RR), past editorial roles at *Systematic Reviews* (JM, SB), authored the Cochrane Handbook guidance on which InSynQ is based [3, 6, 16] (JM, SB, RR) and coauthored reporting guidelines for evidence synthesis, including PRISMA [11], SWiM [14], and PRIOR [21] (JM, SB, RR).

### 2.3 Assessing the reporting of the “PICO for each synthesis” in published systematic reviews

Before commencing work on InSynQ, we examined the extent to which the PICO for each synthesis was clearly and completely reported in a randomly selected sample of 100 systematic reviews of public health and health interventions (presented in full elsewhere [17]). We investigated:

- the extent to which groups were identified and fully specified for each PICO component (e.g., labelled (named), defined in sufficient detail to replicate decisions, rationale for the grouping);
- whether there was a stated plan for each group in the synthesis (e.g., as the basis of comparisons, or to explore possible explanations for inconsistent intervention effects); and
- whether the planned groups were ultimately used in the review's analysis.

In brief, we found that many systematic reviews used PICO characteristics to group studies for synthesis, but reporting was incomplete and variable. It was often not possible to identify the synthesis questions or any definitions (or criteria) that authors had used to decide which studies were eligible for each synthesis.

### 2.4 Assessing the coverage and content of existing reporting guidance

We reviewed existing reporting guidance for systematic reviews to examine the coverage of key concepts and identify potentially relevant items for inclusion or adaptation in InSynQ. The primary sources were the PRISMA 2020 Statement [11], which is the main reporting guidance for systematic reviews, and the Methodological Expectations of Cochrane Intervention Reviews (MECIR) [15], which covers both reporting and conduct specifically for Cochrane reviews. We also examined the PRISMA extension for systematic reviews including network meta‐analysis (PRISMA‐NMA [13]), and the guidance for reviews using synthesis without meta‐analysis (SWiM [14]). All items and content relating to the reporting of review and synthesis questions were collated and considered for inclusion in InSynQ.

### 2.5 Development of initial draft checklist and guide

During the initial drafting of InSynQ, the core team met regularly to discuss content, format and potential templates for the tool and guidance. Our goal was to draft a tool that was brief, practical, and useful to authors, editors, and peer reviewers of systematic reviews, accompanied by sufficient explanation and examples to support implementation. Various options were considered and prototypes developed for discussion with the core team before deciding to proceed with the structure used in PRISMA 2020. An initial list of items for inclusion in InSynQ was drafted based on content from Chapters 2, 3, and 9 of the Cochrane Handbook [3, 6, 16] (specifically tables outlining a process for planning intervention, outcome, and population groups for synthesis in Chapter 3), the data collection tool used in our study of reporting the “PICO for each synthesis” [17] and existing reporting guidance [11, 12, 13, 14, 15]. Feedback on the draft structure and list of items for InSynQ was sought from stakeholders at a consultation meeting (see Section 2.6).

We then drafted content for each item following the structure used in PRISMA 2020 [11, 12]. For each item this included:

- a list of elements to be met in reporting;
- an explanation;
- a list of related guidance and resources; and
- examples of complete and incomplete reporting.

For each item, elements were classified as: *essential* elements that should always be reported (although some are conditionally applicable to reviews with specific features, e.g., reviews including multicomponent interventions); and *additional* elements that would enhance reporting. The examples were largely drawn from reviews of public health and health systems interventions in line with the focus of the program of work funded by Cochrane.

Before circulating the draft guide for feedback, we wrote introductory information about the purpose of InSynQ, its format and structure, who the tool was intended for, the stages of the review to which it was relevant, and how the content relates to existing guidance. The guide also included a two‐page checklist template, listing the items and elements in tabular form, for use as a stand‐alone reporting checklist.

### 2.6 Consultation meetings

We sought feedback from members of the Cochrane community on drafts of the checklist and guide at two online meetings. At the first meeting, members of the core team presented the draft list of items and the proposed structure of the checklist and guide. Fourteen Cochrane members who had previously responded to a call for expressions of interest in the project were invited to attend. Participants at the 1‐h meeting included methodologists, editors, authors with expertise in complex public health reviews and staff of the Cochrane Methods Support Unit (who provide support, peer review, and training on methods to Cochrane authors). Feedback was summarised from notes taken at the meeting and written questions and comments contributed by participants through the meeting chat function, and considered when drafting content for each item and background material.

For the second consultation, members of the core team presented the draft checklist and guide at a web clinic convened by the Cochrane Methods Support Unit [22]. These web clinics aim to provide practical methods support, are open to anyone, and are advertised via the Cochrane website and email newsletters. Participants were invited to discuss selected items from InSynQ in small breakout groups, and then provide feedback for discussion in the large group. Feedback was summarised from notes taken during the meeting and the questions and comments contributed in writing by participants through the meeting chat function. For those unable to attend, the recording of the session was made available on the Cochrane website [22]. Participants were invited to contribute further feedback via an online evaluation survey (see Section 2.7).

### 2.7 Evaluation survey

We sought detailed feedback on the draft checklist and guide through an online evaluation survey. Ethics approval for the survey was received from the Monash University Human Research Ethics Committee (project 32014). An open call for feedback was disseminated via Cochrane newsletters, social media channels, and the Task Exchange website (an online platform for volunteers seeking opportunities to assist in the production of systematic reviews and guidelines, since renamed as Cochrane Engage [23]). Any individual with experience as an author or editor of at least one systematic review was eligible to participate, whether they were Cochrane contributors or not. Recipients could share the invitation through their institutional communication channels and with other interested individuals. In addition, email invitations were sent to 35 methodologists, editors, and experienced systematic review authors, selected for their expertise and interest in the field, using publicly available email addresses.

The online evaluation survey was hosted using Qualtrics [24] (see Supporting Information S1: File 1) between March and May 2022, with responses accepted after this period for those who requested more time. Participant consent was confirmed at the start of the survey, and participants were not required to provide personal or identifying information, although they had the option to provide a name and contact email to receive updates about the project and be acknowledged for their contribution.

A structured survey was used to collect feedback on completeness, content (overall and per item), clarity, and usability. Participants could opt to complete all survey questions, or a subset of questions that excluded those relating to individual items. Optionally, participants were invited to pilot the checklist using either a completed or in‐progress protocol or review of their own choice, and provide additional comments based on that experience, including whether the checklist had indicated changes that could be made to improve reporting in the review (see piloting instructions in Supporting Information S1: File 1).

### 2.8 Revision of draft checklist and guide

Comments received through the consultation meetings and evaluation survey were collated, and options for addressing feedback were proposed and discussed by members of the core team. In weighing up options, we considered whether the feedback indicated an issue with the content or clarity of an item, a broader conceptual issue, or a concern about the feasibility of applying the tool. For broader conceptual issues, we considered whether these were best addressed in the introduction, the explanation and elaboration, additional examples, or reference to guidance in the Cochrane Handbook.

Once complete, the revised draft was reviewed by the wider project group and submitted to Cochrane for consideration for official endorsement as Cochrane methodological guidance. This process is coordinated by Cochrane's Methods Executive and involves collating feedback from relevant stakeholders on acceptability and implications for implementation, including from Cochrane's Editorial Board, Groups that produce Cochrane reviews, Methods Groups, and the Evidence Synthesis Development team and Methods Support Unit within Cochrane's central team. The Methods Executive makes a recommendation based on the feedback to Cochrane's Editor in Chief [25]. If endorsed, Cochrane coordinates dissemination to Cochrane members and authors of Cochrane reviews.

## 3 RESULTS

### 3.1 Coverage and content of existing reporting guidance

Table 1 lists potentially relevant items or elements from existing reporting guidance and indicates how these were used in InSynQ. The sources include items or elements related to reporting research objectives/questions, comparisons, groups used in the synthesis, and outcome specification. These sources do not address all aspects of reporting that were identified as incomplete in our study of published reviews, nor do they provide examples of complete reporting beyond those related to grouping. While PRISMA 2020 and SWiM have reporting elements related to grouping, neither source distinguishes the concept of the PICO for each synthesis from the PICO for the review. The items and their associated explanations reflect this lack of distinction, providing limited coverage of elements needed to fully specify the PICO for each synthesis.

**Table 1:** Items and elements relevant to InSynQ from existing reporting guidelines for systematic reviews of interventions.

Item no.	Section and topic	Source items or elements considered	Use in InSynQ^a^
**PRISMA 2020 Statement—Checklist [** 11 **] and Explanation and elaboration [** 12 **]**			
4	Objectives	Provide an explicit statement of all objective(s) or question(s) the review addresses, expressed in terms of a relevant question formulation framework. If the purpose is to evaluate the effects of interventions, use the Population, Intervention, Comparator, Outcome (PICO) framework or one of its variants, to state the comparisons that will be made.	Informed items 5 and 6
5	Eligibility criteria	Specify any groups used in the synthesis (e.g., intervention, outcome, and population groups) and link these to the comparisons specified in the objectives (PRISMA item #4).	Basis of items 1 and 2
10a	Data items (outcomes)	List and define the outcome domains and time frame of measurement for which data were sought.	Basis of item 2
13a	Synthesis methods (eligibility for synthesis)	Describe the processes used to decide which studies were eligible for each synthesis (e.g., tabulating the study intervention characteristics and comparing against the planned groups for each synthesis [PRISMA item #5])	Included verbatim as item 9
13c	Synthesis methods (tabulation and graphical methods)	If studies are ordered or grouped within tables or graphs based on study characteristics (e.g., by size of the study effect, year of publication), consider reporting the basis for the chosen ordering/grouping.	Informed items 3 and 4
13e	Synthesis methods (methods to explore heterogeneity)	If subgroup analysis or meta‐regression was performed, specify for each: which factors were explored, levels of those factors, and which direction of effect modification was expected and why (where possible). If other methods were used to explore heterogeneity because data were not amenable to meta‐analysis of effect estimates (e.g., structuring tables to examine variation in results across studies based on subpopulation), describe the methods used, along with the factors and levels.	Informed items 1, 2, 3, 4 and 7
20b	Results of syntheses (results of statistical syntheses)	Report results of all statistical syntheses described in the protocol and all syntheses conducted that were not prespecified.	Informed items 10 and 11
24c	Registration and protocol (amendments)	Report details of any amendments to information provided at registration or in the protocol, noting: (a) the amendment itself; (b) the reason for the amendment; and (c) the stage of the review process at which the amendment was implemented.	Informed of items 10 and 11
**SWiM Reporting Guideline** [14]			
1a	Grouping studies for synthesis	Provide a description of, and rationale for, the groups used in the synthesis (e.g., groupings of populations, interventions, outcomes, study design).	Basis of items 1, 2, 3 and 7
1b		Detail and provide rationale for any changes made subsequent to the protocol in the groups used in the synthesis.	Basis of item 10
8	Reporting results	For each comparison and outcome, provide a description of the synthesised findings, and the certainty of the findings. Describe the result in language that is consistent with the question the synthesis addresses, and indicate which studies contribute to the synthesis.	Basis of item 11
**PRISMA‐NMA Extension Statement** [13]			
6	Eligibility criteria	Specify study characteristics (e.g., PICOS, length of follow‐up) and report characteristics (e.g., years considered, language, publication status) used as criteria for eligibility, giving rationale. Clearly describe eligible treatments included in the treatment network, and note whether any have been clustered or merged into the same node (with justification).	Informed items 1, 2, 4 and 5
**Cochrane MECIR** [15]^b^			
C2	Predefining objectives	Define in advance the objectives of the review, including participants, interventions, comparators, and outcomes (PICO).	Informed item 6
PR5	Main objective	State the main objective, where appropriate in a single concise sentence.	Informed item 6
PR6	Secondary objectives	State explicitly (as secondary objectives) any specific questions being addressed by the review, such as those relating to particular participant groups, intervention comparisons or outcome.	Informed item 6
PR15	Outcome measures of interest	Define relevant outcome measures and time points for measurement, and any hierarchy for choosing among them.	Informed item 2
PR36	Subgroup analysis	If subgroup analysis (or meta‐regression) are planned, state the potential effect modifiers with rationale for each.	Informed items 1 and 7
R82	Prespecified outcomes	Report synthesis results for all prespecified outcomes, irrespective of the strength or direction of the result. Indicate when data were not available for outcomes of interest, and whether adverse effects data were identified.	Informed item 11
R107	Changes from the Protocol	Explain and justify any changes from the protocol (including any post hoc decisions about eligibility criteria or the addition of subgroup analyses).	Informed item 10
R108	Methods not implemented	Document aspects of the protocol that were not implemented (e.g., because no studies, or few studies, were found) in the section “Differences between protocol and review,” rather than in the Methods section.	Informed item 10

^a^ Basis = expansion or elaboration of an item or element; Informed = considered when writing the explanation.

^b^ Within the Methodological Expectations for Cochrane Intervention Reviews, items beginning with C, PR, and R relate to conduct, protocol reporting, and review reporting standards, respectively.

### 3.2 Initial draft checklist and guide

The initial draft of the checklist comprised 10 items. Each item was presented with a list of essential elements (with optional additional elements), an explanation, guidance on where to report the item in a systematic review, and links to relevant guidance and other resources. An example of the format used for each Item using the revised structure (following feedback, see Section 3.3) is in Box 2.

Box 2 **Example item from InSynQ**

**Table:** 

**Item 1. Specify population and intervention groups to be used in the synthesis** **Explanation** In any synthesis, studies with similar features are grouped to examine intervention effects, or factors that modify effects. Providing a clear label (name) and definition for each of the intervention and population groups to be used in the synthesis will help readers understand the planned structure of the synthesis and assess whether the proposed groupings will appropriately address the objectives of the review. Such description will also help ensure methods are transparent and that decisions about which studies contribute to each synthesis are replicable. Reporting of groups used in the synthesis should: be explicit (not implied); be presented as a complete list (not limited to examples); avoid using labels without definition (such as “usual care” without stating “as defined by trialists” or providing criteria to define usual care); cover all syntheses (i.e., comparisons, subgroups), structured summaries (e.g., text and results tables), and summaries of the review (e.g., Summary of Findings tables); cover any plans to group at more than one level to address both a broad question (e.g., what is the effect of “any exercise intervention”) and more specific questions (e.g., what is the effect of: “weight‐bearing exercise,” “nonweight‐bearing exercise” …); and cover any contingencies, such as plans to group more broadly if there are insufficient studies to address specific questions (e.g., a plan to group “all forms of exercise” if there are too few studies to examine specific types of exercise). **Where to report**Groups may be specified in different sections of the review (e.g., Background, Methods) and in different formats (e.g., dot point list, descriptive text, boxes or tables, logic models or figures) as long as it is clear to the reader that the specified groups are to be used in the synthesis. See examples. **Essential elements** Label (name) each group. Define each group in enough detail to replicate decisions about which intervention (or population) group(s) each study is eligible for. Where the definitions are based on an established source (e.g., a taxonomy of interventions), it may be sufficient to identify and reference the source. If your review includes studies with multicomponent interventions, specify how these will be defined and grouped for each synthesis. If your review includes inactive comparators (e.g., usual care, no intervention), specify how they will be grouped for synthesis. Describe any plans to group at multiple levels, to address both broad and specific questions. Describe any contingency plans for accommodating the amount of available evidence (e.g. a plan to group more broadly if too few studies to address specific questions). **Additional elements** Consider presenting detailed definitions in boxes or tables. Consider using logic models or figures to provide a visual summary of groups, and the links between different PICO elements and the groups within each.

### 3.3 Feedback from consultation meetings and survey

Six people attended the first consultation at which the draft template and list of items was presented, including experienced authors, editors, and methodologists based in the United Kingdom and Australia. Participants were generally supportive of the checklist items and the overall format, and emphasised the helpfulness of examples. No suggestions were received to add or remove items. Prompted by general discussion points, we drafted introductory text explaining which InSynQ items applied to protocol stage (items 1–8) and completed review stage (all items) and outlining the types of reviews for which the tool could be used. For each item, we added a suggested location in which the required information could be reported in a protocol or review manuscript.

Sixteen people (excluding the authors of this paper) participated in the second meeting, including systematic review authors, editors, and methodologists. Twelve people completed the survey (eight from direct email sent to 35 people; four from the public call). Survey respondents all had experience with systematic reviews in one or more roles, specifically as authors (8/12, 67%), editors (4/12, 33%), methodologist/statisticians (5/12, 47%), or other roles (2/12, 17%). Two additional people provided comments by email. One respondent (RR, a member of the authorship team) piloted the checklist on two existing systematic review manuscripts—one protocol and one completed review.

Table 2 summarises suggestions for improvement received through the second consultation meeting, the survey or email, and how they were addressed in revising InSynQ. In addition, survey respondents provided feedback on their overall impression of the checklist and guide. All respondents stated that they believed the checklist would help them to set up the synthesis questions in their own review, or to appraise the synthesis questions in someone else's review. All would recommend the checklist to others. Most respondents (8/12, 83%) felt that the checklist would be extremely or somewhat easy to use. The use of examples was also endorsed. No respondents suggested removing items. Three additional items for inclusion in the checklist were suggested.

**Table 2:** Summary feedback on InSynQ and how it was addressed.

Feedback		Source	How addressed
Conceptual			
The concept of PICO for synthesis was unfamiliar, and the concepts of “groups” (especially outcomes and methodological groups) and grouping at multiple levels were confusing to some.		Meeting 2; Survey	Added box to introduction to illustrate concept of PICO for each synthesis and options for grouping interventions at different levels. Revised items 1 and 2; integrated examples into elements.
The approach taken in InSynQ represents a fundamental shift wherein the methodology for developing the synthesis questions is formalised at the review planning stage.		Pilot	Revised introduction to clarify expectations. Plan to discuss new methodological approach in other papers.
Content and clarity of items			
Item 1	Clarify the concepts of PICO for synthesis, groups, and grouping at multiple levels.	Meeting 2; Survey	Revised text (cross‐referenced to boxed example in introduction); integrated examples into elements.
Item 2	Clarify the concepts of PICO for synthesis, groups, and grouping at multiple levels.	Meeting 2; Survey	Revised text; integrated examples into elements.
Item 4	Clarify the meaning of “role in synthesis”.	Survey	Edited explanation and examples for clarity and consistency.
Item 5	Selection and prioritisation of comparisons should be informed by clinically important questions.	Meeting 2; Email	Added explanatory text and element about specifying the order of importance of comparisons.
[New] Item 8	Add item on how consultation with consumers and other stakeholders informed the synthesis.	Meeting 2; Core team discussion	Added item on consultation with consumers and other stakeholders. Content reviewed by two researchers with expertise in patient and public involvement in SRs.
Other suggested items	Add item requiring review of search strategy after questions finalised.	Survey	No change. Steps subsequent to question formulation were beyond the scope of InSynQ.
	Add item on risk of bias.	Surveys	No change (as per search strategy).
Examples	Suggestions for optimal location of examples.	Survey; Pilot	Added brief examples to some item explanations and elements to illustrate concepts and terms. Retained detailed examples at end of the guide; hyperlinked example from items.
General	Use simple and concise language.	Survey	Language was reviewed throughout.
Structure and feasibility of application			
Clarify whether the tool is for reporting, development, or both.		Meeting 2; Pilot	Edited title/introduction: InSynQ intended for question development and reporting. MECIR reporting standards replaced with MECIR conduct standards.
Concerns that these reporting requirements will lengthen text and increase workload.		Email; Pilot	Added section to introduction addressing concerns about workload and length of text.
Add key content to the 2‐page checklist template for quick reference.		Survey; Pilot	Integrated brief examples into elements for some items; no content added to retain length of template at 2 pages.
Suggestions for structure/order of information in Items.		Survey; Pilot	No change; retained familiar format of PRISMA [12] and SWiM [14].

### 3.4 Final list of items and dissemination of InSynQ

The final list of 11 items is in Box 3. The complete InSynQ checklist and guide are available at https://InSynQ.info [26]. InSynQ has been formally endorsed by Cochrane [27].

Box 3. **Final items included in InSynQ checklist and guide**

**Table:** 

1. Specify population and intervention groups to be used in the synthesis 2. Specify outcome groups to be used in the synthesis 3. Give a rationale for the groups 4. Identify the role of each group in the synthesis 5. Specify the pairwise comparisons that will be made between intervention groups 6. Ensure that the Objectives align with the questions addressed in the synthesis 7. Specify methodological groups to be used in the synthesis. 8. Identify how patients, the public, and other stakeholders informed the development of questions to be addressed in the synthesis 9. Describe the processes used to decide which studies were eligible for each synthesis* 10. Identify changes made at review stage to the groups or comparisons reported in the protocol 11. Report the results in accordance with the groups and comparisons specified in the methods

*Reproduced from PRISMA 2020 [12]

## 4 DISCUSSION

In this paper, we describe the development of the InSynQ tool for planning and reporting synthesis questions in systematic reviews of interventions. Planning and articulating the PICO for each synthesis is an instrumental, yet under recognized, part of planning how the synthesis will be conducted. While the Cochrane Handbook provides methods for planning synthesis questions and criteria for grouping studies, Cochrane identified a need for a practical tool to help authors implement this guidance. Underpinned by findings from our study examining the reporting of synthesis questions in published reviews, this provided the impetus to develop InSynQ. To develop InSynQ, we used methods informed by those of the EQUATOR Network [20]. By reporting in detail the methods used, we aim to be transparent about the basis for the selected items and other content, who contributed to the development of the checklist and guide, and its endorsement. While such methods are not always reported in full, doing so helps build an evidence base about the types of methods used to develop guidance on research conduct and reporting [28]. This evidence base should help developers of guidance consider the methods options available, and the circumstances under which particular methods might be selected ahead of others.

### 4.1 Strengths and limitations

InSynQ addresses a gap, identified through our own research and independently by Cochrane, in the availability of practical tools to help authors develop and completely report their synthesis questions in systematic reviews of interventions. Development of the InSynQ checklist and guide followed a structured, consultative process. Content was based on guidance published in the Cochrane Handbook in 2019 [3, 6], examination of existing reporting guidance and a review of practice in published systematic reviews, and an open process to seek and receive feedback in which more than 30 people engaged. The latter was an important step in ensuring that the experience and views of review authors, editors, and methodologists beyond those of the author team were reflected in the first version of InSynQ. However, the timeframe of the project was short and it will be important to review InSynQ in light of feedback as the tool is more widely implemented.

We chose not to undertake a Delphi process for this version of InSynQ. While a Delphi process is commonly used to reach consensus on the items included in reporting checklists [20], achieving consensus requires shared understanding of concepts. The findings from our review of practice in published systematic reviews and discussion during the consultation meetings suggested that the concept and reporting elements associated with the PICO for each synthesis are relatively new to many. For this reason, we prioritised dissemination of the tool to enable more widespread experience in applying these concepts in practice to be gained by authors, editors and others.

### 4.2 Implications for systematic review practice

InSynQ provides a practical tool for authors, editors, and peer reviewers of systematic reviews of interventions, supporting the planning and reporting of the question, comparison, and eligibility criteria for each synthesis (i.e., the “PICO for synthesis”). A critical first step for implementation is to report the PICO for each synthesis in completed reviews. Prespecifying the PICO for each synthesis poses additional challenges because of the need to anticipate and plan for diversity in the PICO questions addressed by eligible studies.

For those producing Cochrane reviews, the process will be enabled by enhancements to Cochrane's systematic review production software, RevMan Web [29]. The Cochrane Handbook guidance [3, 6, 16], the InSynQ checklist provide a methodological underpinning for these enhancements. The enhancements are embedded in the study‐centric data management features of RevMan Web and prompt authors to define the PICO for each synthesis at the protocol stage, and then structure their synthesis by defining comparisons, intervention groups, the interventions eligible for each group, and other PICO components. Setting up the review criteria and PICO for each synthesis within the software facilitates data extraction, enables data imports, and automatically generates meta‐analyses in accordance with the planned comparisons [30].

We expect the InSynQ checklist and guide [26] will be refined as users provide feedback based on their experience applying the checklist in practice. To enable changes to be disseminated, the current version of the checklist and guide are made available on a dedicated website (https://InSynQ.info). Other implementation activities for InSynQ include dissemination to Cochrane members, incorporation into training resources for authors of systematic reviews, and presentation to members of systematic review‐producing organisations, including Cochrane, the Campbell Collaboration, and JBI. Additional resources that may help to support the implementation of InSynQ include structured templates for systematic reviews and protocols that incorporate reporting against the InSynQ items, and expansion of the set of examples to encompass a wider range of questions, including clinical questions. Further work could investigate whether the approach taken in InSynQ would be useful for planning and reporting other types of questions addressed in systematic reviews, and what would be needed to adapt or extend the tool where different question frameworks are used, such as reviews of exposures (“PECO”) [31], diagnostic test accuracy (“PIT”) [32], or prevalence (“CoCoPop”) [33].

## 5 CONCLUSION

The InSynQ checklist and guide for reporting of intervention synthesis questions was developed from guidance in the Cochrane Handbook, an empirical study of the reporting of synthesis questions in published systematic reviews, an assessment of existing reporting guidance, and a formal process of consultation and feedback. In reporting the methods used to develop InSynQ, we aim to provide transparency about the basis of the tool for users and contribute to the evidence base for methods used to develop conduct and reporting guidelines. The InSynQ tool is designed to support those involved in the production of systematic reviews to plan and completely report their synthesis questions and structure, increasing the transparency and reproducibility of decisions. In turn, this is expected to facilitate better defined and more focused reviews, enhancing the clarity of planned syntheses and reviews for decision makers.

## AUTHOR CONTRIBUTIONS

**Miranda S. Cumpston**: Data curation; formal analysis; funding acquisition; investigation; methodology; project administration; writing—original draft; writing—review and editing. **Joanne E. McKenzie**: Conceptualization; funding acquisition; investigation; methodology; supervision; writing—review and editing. **Rebecca Ryan**: Investigation; methodology; writing—review and editing. **Ella Flemyng**: Investigation; project administration; writing—review and editing. **James Thomas**: Investigation; writing—review and editing. **Sue E. Brennan**: Conceptualization; funding acquisition; investigation; methodology; project administration; supervision; writing—original draft; writing—review and editing.

## CONFLICT OF INTEREST STATEMENT

The authors declare no conflict of interest.

## PEER REVIEW

The peer review history for this article is available at https://www.webofscience.com/api/gateway/wos/peer-review/10.1002/cesm.12036.

## ETHICS STATEMENT

Ethics approval for the evaluation survey included in this paper was received from the Monash University Human Research Ethics Committee (project 32014).

## Supporting information

Supporting information.

## Data Availability

Data available on request due to privacy/ethical restrictions.
