# Supplementary material for: Development of the InSynQ checklist: A tool for planning and reporting the synthesis questions in systematic reviews of interventions
Source: Cochrane Evid Synth Methods. 2023 Dec 12;1(10):e12036. doi: 10.1002/cesm.12036 (PMC11795942; doi:10.1002/cesm.12036)
Supplement: Supplementary file 1 — Supporting information. [file CESM-1-e12036-s001.pdf]

Supplementary File 1

Contents

Section A. SynQ (Synthesis Questions) checklist and guide: feedback survey 1
Section B: SynQ (Synthesis Questions) reporting checklist and guide: Instructions for piloting...... 18

Section A. SynQ (Synthesis Questions) checklist and guide: feedback survey

[Note: At the time of the survey, the InSynQ checklist and guide was referred to with the slightly different abbreviation, 'SynQ'.]

SynQ (Synthesis Questions) checklist and guide: feedback survey
Evaluation of a checklist and guide for defining the questions to be addressed in the synthesis of health intervention reviews

What does the survey involve?

The SynQ (Synthesis Questions) reporting checklist and guide has been drafted for defining the synthesis questions to be addressed in systematic reviews of interventions. Development of SynQ was supported by Cochrane and led by researchers at Cochrane Australia and the Methods in Evidence Synthesis Unit at Monash University.

Authors and editors of systematic reviews are now invited to provide feedback on the draft checklist and guide. To participate, you will be asked to:

- Read the SynQ checklist and guide (approx. 30 minutes). Download the SynQ checklist and guide here.
- Optionally, you may choose to pilot the checklist using a review that you are familiar with, and provide additional feedback on your experience applying the checklist items (this may take up to two hours, depending on your choice of approach). Download instructions for piloting here.
- Complete this online survey to provide your feedback (approx. 20 minutes).

If possible, we would like to receive the first round of feedback by **Thursday, 14 April 2022**. For those who wish to provide feedback after this date, the online form will remain open until Tuesday, 31 May 2022.

Confidentiality and ethics

Feedback received through this survey will be used to inform the next version of SynQ. Aggregate, de-identified summaries of the feedback received may be included in journal publications, conference presentations and a PhD thesis. If you wish, you may complete this survey anonymously. Note that if your contact details are not included in your response, it may not be possible to identify and withdraw your responses after they have been submitted, if you wished to do so.

If you have any ethics concerns or complaints about the conduct of the survey, please contact the Monash University Human Research Ethics Committee at muhrec@monash.edu (Project ID 32014).

Chief Investigators: Dr Sue Brennan, School of Public Health and Preventive Medicine, Monash University. Phone: +61 3 9903 0366. Email: sue.brennan@monash.edu; and
Associate Professor Joanne McKenzie, School of Public Health and Preventive Medicine, Monash University. Phone: +61 3 9903 0380. Email: joanne.mckenzie@monash.edu.

Co-Investigators: Miranda Cumpston (PhD candidate, School of Public Health and Preventive Medicine, Monash University); and Dr Rebecca Ryan (Centre for Health Communication & Participation, La Trobe University).

Consent

I have been asked to take part in the Monash University research project specified above. I have read and understood the Explanatory Statement (download Explanatory statement here) and I hereby consent to participate in this project.

	Yes	No
Taking part in an online survey.	☐	☐
The data that I provide during this research may be used in future research projects (with appropriate ethics approval and de-identification).	☐	☐
Inclusion of my name (and affiliation) in the Acknowledgements section of any papers reporting results from this project, in a list of participants acknowledged for contributing expert knowledge.	☐	☐
Inclusion of my name (and affiliation) on a slide in presentations or meetings about this project, in a list of participants acknowledged for contributing expert knowledge.	☐	☐

Instructions for completing the survey

This aim of this survey is to gather feedback on the draft SynQ reporting checklist and guide. The survey will allow you to submit feedback in as much detail as you feel comfortable providing.

Before starting the survey, ensure that you have read the SynQ checklist and guide. If you have chosen to pilot the checklist on a specific protocol or review, you should also complete the piloting process before responding to the survey.

You will have the option to provide:

- general feedback on SynQ as a whole; and/or
- specific feedback on each item in the checklist and its associated explanation; and/or
- feedback after piloting the checklist on a systematic review with which you are familiar.

You can save your responses, then close the browser and return to the survey later. If possible, we would like all feedback to be submitted by **Thursday, 14 April 2022**. The survey is compatible with mobile devices.

The complete survey can be downloaded here.

Where can I get further information?

If you would like to discuss the SynQ checklist or the feedback survey, please contact Miranda Cumpston (miranda.cumpston@monash.edu).

A. Preliminary information

Which of these roles describes you best?
Choose all that apply

- Author (completed 1-2 reviews)
- Author (completed >2 reviews)
- Editor
- Methodologist/statistician
- Other (please specify)____

How did you hear about this feedback survey on the SynQ checklist and guide?

- I was invited by the developers to provide feedback
- From a colleague
- From a newsletter or social media announcement
- On Cochrane Task Exchange
- Other (please specify) ____

Contact information *(optional)*

- Name:____
- Email:____

B. Feedback on individual checklist items

This section asks for feedback on individual items in the checklist, and the associated explanation and examples. Alternatively, you may skip ahead to give overall feedback on the checklist and guide as a whole.

Would you like to give feedback on individual items in the checklist?

- Yes
- No, I would like to proceed to giving overall feedback

Which item(s) would you like to give feedback on? *(Select all that apply)*

- All items
- Item 1
- Item 2
- Item 3
- Item 4
- Item 5
- Item 6
- Item 7
- Item 8
- Item 9

- Item 10

Item 1. Specify population and intervention groups to be used in the synthesis

Essential elements

- Label each group.
- Define each group in enough detail to replicate decisions about which intervention (or population) group(s) each study is eligible for. Where the definitions are based on an established source (e.g. a taxonomy of interventions), it may be sufficient to identify and reference the source.
- If your review includes studies with multi-component interventions, specify how these will be defined and grouped for each synthesis.
- If your review includes inactive comparators (e.g. usual care, no intervention), specify how they will be grouped for synthesis.
- Describe any plans to group at multiple levels, to address both broad and specific questions.
- Describe any contingency plans for accommodating the volume of available evidence.

Additional elements

- Consider presenting detailed definitions in boxes or tables.
- Consider using logic models or figures to provide a visual summary of groups, and the links between different PICO elements and the groups within each.

Do you agree with the proposed item, including the essential and additional elements?

- Agree
- Agree, but some changes to the wording are required *(please explain)*
- Disagree (*please explain)*
- Don't know

Explanation

Providing a clear label and definition of each of the intervention and population groups to be used in the synthesis will help readers understand the planned structure of the synthesis and assess whether the proposed groupings will appropriately address the objectives of the review. Such description will also help ensure methods are transparent and that decisions about which studies contribute to each synthesis are replicable.

Reporting of groups used in the synthesis should:

- be explicit (not inferred);
- be presented as a complete list (not be limited to examples);
- avoid using labels without definition (such as ‘usual care’ without saying ‘as defined by trialists’ or providing criteria to define usual care);
- cover all syntheses (i.e. comparisons, subgroups), structured summaries (e.g. text and results tables), and summaries of the review (e.g. Summary of Findings tables);
- cover any plans to group at more than one level in order to address both a broad question (e.g. what is the effect of ‘any exercise intervention’) and more specific questions (e.g. what is the effect of: ‘weight bearing exercise’, ‘non-weight bearing exercise’ …); and
- cover any contingencies, such as plans to group more broadly if there are insufficient studies for narrower groupings required to address specific questions (e.g. a plan to group

‘all forms of exercise’ if there are too few studies to examine effects of specific types of exercise).

Do you agree with the *content* of the proposed explanation?

- Agree
- Agree, but some additional rationale could be provided (please explain)____
- Disagree (please explain)____

Any additional comments: ____

Item 2. Specify outcome groups to be used in the synthesis

Essential elements

- Label each outcome group.
- Define each outcome group (i.e. the ‘what is being measured’) in enough detail to enable eligible outcomes from each included study to be categorised.
- Specify the measurement methods or tools/scales that provide an appropriate assessment of the domain (i.e. the ‘how the outcome is measured’).
- Specify the time frame of the outcome group (i.e. the ‘when the outcome is measured’) (e.g. up to 12 weeks).
- Describe any plans to group outcomes at multiple levels, to address both broad and specific questions.
- Describe any contingency plans for accommodating the volume of available evidence (e.g. a plan to group ‘any health behaviour’ outcome if there are too few studies to examine effects on specific health behaviours).

Do you agree with the proposed item, including the essential elements?

- Agree
- Agree, but some changes to the wording are required *(please explain)*
- Disagree *(please explain)*
- Don't know

Explanation

Fully specifying outcome groups (domains) to be used in each synthesis allows readers to understand the specific research questions addressed in the review (i.e. the review objectives). For example, whether a review of new generation antidepressants will examine the effects of antidepressants on ‘depression at any time point’, ‘depression in the short term (up to 12 weeks)’, ‘depression in the longer term (up to 12 months)’, or all three outcome groups. Moreover, providing sufficient detail for each outcome group allows readers to assess the potential for the groups to have been driven by the results of the available studies. This detail also aids in replication of the decisions about which studies contribute to each synthesis. This is particularly important for outcomes assessed by measurement scales, where different scales are used across

the studies, and decisions need to be made as to whether the scales provide a measure of the review outcome, and thus should contribute to the synthesis.

A scenario to avoid is providing a list of outcomes in the ‘Types of outcome measures’ section, without clearly specifying the level at which the outcomes will be grouped for synthesis. For example, by having a heading ‘depression’ under which is listed ‘Hamilton Depression Rating Scale (HAM-D)’, ‘Montgomery-Asberg Depression Rating Scale (MADRS)’, ‘Children’s Depression Rating Scale (CDRS-R)’, it is not clear if the planned group for synthesis is ‘depression’, or if separate syntheses will be undertaken for each of the measurement scales.

Do you agree with the *content* of the proposed explanation?

- Agree
- Agree, but some additional rationale could be provided *(please explain)*
- Disagree *(please explain)*
- Don't know

Any additional comments:____

Item 3. Give a rationale for the groups

Essential elements

- For each PICO element, describe the basis for grouping with a rationale.
- If grouping is based on an existing system (e.g. a published conceptual framework, taxonomy or core outcome set, or other reviews), identify and reference the source, noting any adaptations made for the review.

Additional elements

- Consider presenting a logic model or figure to provide a visual summary of the links between the different PICO elements and the groups within each, and the mechanisms (pathways) of action.

Do you agree with the proposed item, including the essential and additional elements?

- Agree
- Agree, but some changes to the wording are required *(please explain)*
- Disagree *(please explain)*
- Don't know

Explanation

The groups used in a synthesis can be based on a variety of characteristics (e.g. intervention content, purpose or mode of delivery; outcome measurement method or timing; population characteristics associated with inequitable health outcomes). Decisions about grouping may be underpinned by a theoretical rationale (e.g. the possible mechanism(s) of action), an existing system, empirical evidence, current practice, the priorities of decision-makers or other factors. Decisions should also account for practical issues that impact on the synthesis. For example, the

volume of available evidence will determine whether it is possible to answer specific or more general questions (reflected in narrow or broader groupings).

Reporting the basis for the groups used in the synthesis and the associated rationale helps the reader understand what options were considered and why particular decisions were made. Such decisions may seem self-evident to the review authors, but typically involve assumptions that will not be evident to readers of the review unless reported. This includes the special case where all types or variants of a seemingly diverse category of interventions (or other PICO element) are included in a single broad group for synthesis (e.g. any exercise, any psychosocial intervention, any health behaviour outcome). Providing an explicit rationale for the groups used in the synthesis enables readers to verify that the approach aligns with the review objectives, rather than being driven by available studies. This is especially important for analyses that examine whether particular characteristics modify the intervention effects (e.g. intervention intensity or setting), the credibility of which is increased by a priori specification of a scientific rationale for the hypothesised direction of effect modification.

Do you agree with the *content* of the proposed explanation?

- Agree
- Agree, but some additional rationale could be provided (*please explain*)
- Disagree (*please explain*)
- Don't know

Any additional comments:____

Item 4. Identify the role of each group in the synthesis

Essential elements

- Identify which of the specified groups will form the basis of comparisons and any groups that will be used to stratify studies within the comparisons.
- If applicable, identify which of the specified groups will be used to explore possible causes of variation in the effects of an intervention (e.g. in subgroup analyses or meta-regression).
- If applicable, identify which of the specified groups will be used in sensitivity analyses to test the robustness of the findings to the decisions or assumptions made in the analysis.
- Identify any other roles the specified groups have in the synthesis or summary (e.g. to structure text, tables or figures).
- If a logic model or figure is used to display groups, be explicit about the role of these groups in the synthesis.

Do you agree with the proposed item, including the essential elements?

- Agree
- Agree, but some changes to the wording are required (*please explain*)
- Disagree (*please explain*)
- Don't know

Explanation

Each of the specified groups should be explicitly linked to the synthesis so that readers can identify whether the groups will be (are) used:

- as the basis of comparisons (i.e. intervention groups and outcomes specified for each meta-analysis or other synthesis);
- to structure text, tables or figures (e.g. to stratify studies in a forest plot according to population or intervention characteristics, or by methodological characteristics);
- to explore possible causes of variation in the effects of an intervention (i.e. in subgroup analyses or meta-regression used to explore possible causes of statistical heterogeneity);
- to assess the robustness of the results of the synthesis to the decisions or assumptions made in the analysis (i.e. in sensitivity analyses); or
- to convey the scope of the review, for example the range of populations or interventions included in the review, with no intended role in the synthesis.

Linking groups to the synthesis provides clarity about the structure of the synthesis, especially in the common scenario where specific interventions or outcomes are listed under broad categories. Take for example a review where the authors list interventions under the broad categories ‘relational approaches’ (e.g. parent-child interventions, family-focused interventions) and ‘mind-body approaches’ (e.g. group music therapy, trauma-focused art therapy, yoga). Both the broad category and the specific interventions within could have roles in the synthesis.

Reporting the role of these groups in the synthesis is needed so that readers can ascertain whether:

- studies will be grouped by the broad categories for comparison, with or without stratification by the specific types of intervention;
- studies will be grouped by the specific interventions in separate comparisons, without further aggregation (or drawing conclusions) at the level of the broad category;
- the broad categories will be used only to structure the text; or
- the specific interventions are mentioned only to convey the scope of interventions eligible for the review.

Do you agree with the *content* of the proposed explanation?

- Agree
- Agree, but some additional rationale could be provided (*please explain*)
- Disagree (*please explain*)
- Don't know

Any additional comments:____

Item 5. Specify the pairwise comparisons that will be made between intervention groups

Essential elements

- Specify all of the comparisons to be made between intervention groups.
- Specify whether co-interventions will be included in the same or separate comparisons.
- Provide a rationale for the selected comparisons (when these are a subset of all possible comparisons).

Do you agree with the proposed item, including the essential elements?

- Agree
- Agree, but some changes to the wording are required (*please explain*)
- Disagree (*please explain*)
- Don't know

Explanation

Specifying the pairwise comparisons that will be made between intervention groups allows readers to understand the planned syntheses and assess whether they align with the review objectives. While in reviews with only two intervention groups (e.g. cognitive behaviour therapy (CBT), wait-list (WL) control) the pairwise comparison is self-evident (CBT versus WL); this is not the case when there are multiple intervention groups. For example, in a review including the intervention groups CBT, behaviour therapy (BT), cognitive therapy (CT), and WL, there are six possible comparisons; however, only a subset of comparisons may be of interest in the review (e.g. CBT versus WL, BT versus WL, CT versus WL). Pre-specifying the comparisons reassures readers that those presented were planned, and not just those showing 'interesting' or 'favourable' results.

In the special case of co-interventions, where the same supplementary intervention is delivered in both intervention groups (e.g. CBT + antidepressant versus WL + antidepressant), it is important to specify whether these studies will contribute to the same comparison (e.g. CBT versus WL), or as a different comparison. If the first option is taken, an additional consideration to report is whether there will be subgrouping by the comparisons involving co-interventions and those without.

Do you agree with the *content* of the proposed explanation?

- Agree
- Agree, but some additional rationale could be provided (*please explain*)
- Disagree (*please explain*)
- Don't know

Any additional comments:____

Item 6. Ensure that the Objectives align with the questions defined for the synthesis

Essential elements

- Ensure that the objectives cover the questions addressed in the synthesis in sufficient detail to match the objectives to the corresponding syntheses.
- Use consistent wording (terminology) across all sections of the review where the questions addressed in the synthesis are reported, including in the objectives.

Do you agree with the proposed item, including the essential elements?

- Agree
- Agree, but some changes to the wording are required (*please explain*)

- Disagree (*please explain*)
- Don't know

Explanation

While authors are encouraged to define the objectives of their review (i.e. the questions addressed by the review) before developing detailed PICO criteria, ultimately it is important to ensure that there is alignment between the reporting of the review objectives and the specific questions defined in the PICO for each synthesis. These PICO are articulated through the specification of groups and their role in the synthesis (Items 1 to 5) and can be considered an operationalisation of the review objectives. As a final step in reporting, authors should ensure that the objectives cover the questions (to be) addressed in the synthesis using consistent wording. Doing so enables readers to understand the purpose of the review and match the objectives to the corresponding syntheses and findings.

Take, for example, a review of school based interventions to promote physical activity. After defining the intervention, outcome and population groups to be used in the synthesis, the authors should ensure that their objectives capture the defined groups and how these will be used in the synthesis.

For example, the objectives might be:

- to estimate the pooled effect of school based interventions on physical activity, fitness and body composition and whether this effect is modified by type of intervention and age (children versus adolescents); and
- to estimate the effect of each type of school based intervention (i.e. before or after school physical activity, enhanced physical education classes, school time physical activity, multi-component), compared with usual practice, on physical activity, fitness and body composition.

Note in this example, the authors plan to synthesise at two levels; in the first, examining 'any' school based interventions to promote physical activity versus usual practice, and in the second examining the effects of four specific types of interventions (each need to be defined). We would expect all five intervention groups and their role in the synthesis to be identified, and the same for the outcome and population groups. If the authors had elected to examine the effects of 'any' school based intervention, then we would expect a rationale so that it is clear to the reader that the objectives and synthesis questions align.

Do you agree with the *content* of the proposed explanation?

- Agree
- Agree, but some additional rationale could be provided (*please explain*)
- Disagree (*please explain*)
- Don't know

Any additional comments:____

Item 7. Specify methodological groups to be used in the synthesis

Essential elements

- Provide the basis for grouping with a rationale.
- Label each methodological group.
- Define each methodological group in enough detail to enable classification of studies into groups.
- Describe the role of the methodological groups in the synthesis.

Do you agree with the proposed item, including the essential elements?

- Agree
- Agree, but some changes to the wording are required (*please explain*)
- Disagree (*please explain*)
- Don't know

Explanation

The focus of systematic reviews of interventions is to address research questions about the benefits and harms of clinical and public health interventions; items 1 -5 guide review authors in articulating these. A common secondary focus will be to examine whether the findings from analyses addressing these research questions are robust to methodological factors (e.g. risk of bias (RoB), study design features, outcome assessment methods). Such analyses can reassure readers, and thus provide them with confidence in the findings or, conversely, raise concerns.

Similar to populations, interventions and outcomes, it is important to provide a clear label and definition of each methodological group to be used in the synthesis (e.g. low RoB studies, some concerns / high RoB studies; OR low / some concerns RoB studies, high RoB studies), the basis for grouping with a rationale (item 3), and role of the group in each synthesis (e.g. sensitivity analysis, subgroup analysis) (item 4).

Providing readers with sufficient detail of the methodological factors, groups and role in the synthesis, allows readers to:

- assess whether all important factors have been investigated;
- verify that the analyses are not data driven to obtain a desired result; and
- replicate decisions about which studies contribute to each methodological group.

Do you agree with the *content* of the proposed explanation?

- Agree
- Agree, but some additional rationale could be provided (*please explain*)
- Disagree (*please explain*)
- Don't know

Any additional comments:____

Item 8. [Reproduced from PRISMA 2020 item 13a] Describe the processes used to decide which studies were eligible for each synthesis (such as tabulating the study intervention characteristics and comparing against the planned groups for each synthesis (items 1 to 5))

Essential elements

- Describe the processes to be used to decide which studies were eligible for each synthesis.

Do you agree with the proposed item, including the essential elements?

- Agree
- Disagree (*please explain*)
- Don't know

Explanation

Before undertaking any statistical synthesis (item #13d), decisions must be made about which studies are eligible for each planned synthesis (item #5). These decisions will likely involve subjective judgments that could alter the result of a synthesis, yet the processes used and information to support the decisions are often absent from reviews. Reporting the processes (whether formal or informal) and any supporting information is recommended for transparency of the decisions made in grouping studies for synthesis. Structured approaches may involve the tabulation and coding of the main characteristics of the populations, interventions, and outcomes. For example, in a review examining the effects of psychological interventions for smoking cessation in pregnancy, the main intervention component of each study was coded as one of the following based on pre-specified criteria: counselling, health education, feedback, incentive-based interventions, social support, and exercise. This coding provided the basis for determining which studies were eligible for each planned synthesis (such as incentive-based interventions versus usual care). Similar coding processes can be applied to populations and outcomes.

Do you agree with the *content* of the proposed explanation?

- Agree
- Agree, but some additional rationale could be provided (*please explain*)
- Disagree (*please explain*)
- Don't know

Any additional comments:____

Item 9. Identify changes made at review stage to the groups or comparisons reported in the protocol

Essential elements

- Label and define any groups used in the review that were not reported in the protocol.
- List any comparisons made in the review that were not reported in the protocol.
- Provide a rationale for any changes made during the review to the planned groups or comparisons.

Do you agree with the proposed item, including the essential elements?

- Agree
- Agree, but some changes to the wording are required (*please explain*)
- Disagree (*please explain*)
- Don't know

Explanation

When conducting a review, especially one involving intervention complexity, authors may need to change the groups or comparisons reported in the protocol in order to make best use of available data. Examples include:

- where insufficient studies are found for a planned comparison so a decision is taken to group more broadly (including the decision to include 'any' intervention in a single group);
- where insufficient information is available to decide which studies are eligible for a planned group;
- where a clearly important group has been overlooked at the planning stage; or
- where a clearly important potential effect modifier has been overlooked at the planning stage.

Any changes should be reported with a rationale and the new approach described.

Do you agree with the *content* of the proposed explanation?

- Agree
- Agree, but some additional rationale could be provided (*please explain*)
- Disagree (*please explain*)
- Don't know

Any additional comments:____

Item 10. Report the results in accordance with the groups and comparisons specified in the methods

Essential elements

- Report using the same groups and comparisons as specified in the methods.
- Report using the same group labels.

Do you agree with the proposed item, including the essential elements?

- Agree
- Agree, but some changes to the wording are required (*please explain*)
- Disagree (*please explain*)
- Don't know

Explanation

The reporting of the results of the review should be consistent with the final PICO for each synthesis reported in the objectives and methods. Consistency helps readers navigate the review and enables easy matching of the results to the questions. Consistency can be achieved in a number of ways; for example, by:

- using the same groups and comparisons as specified in the methods;
- using the same group labels (i.e. using consistent terminology);
- presenting the results in the same order as the objectives are presented;
- including headings in the results to indicate which objectives are being addressed.

In the circumstance where syntheses cannot be undertaken, the groups and comparisons specified in the methods can still be used to structure the text of the review, tables and figures, as well summary versions of the review (Summary of Findings Tables, Abstract, Plain language summary).

Do you agree with the *content* of the proposed explanation?

- Agree
- Agree, but some additional rationale could be provided (*please explain*)
- Disagree (*please explain*)
- Don't know

Any additional comments:____

Overall feedback on the SynQ checklist and guide

Does the checklist include all the items needed to facilitate complete and accurate reporting of the questions addressed in systematic reviews?

- Yes
- No
- Don't know

Please describe any items that are missing:____

Did you find the checklist and guide easy to understand?

- Extremely easy
- Somewhat easy
- Neither easy nor difficult
- Somewhat difficult
- Extremely difficult

Overall, do you think the checklist would help you to set up the synthesis questions in your own review, or to appraise the synthesis questions in someone else's review?

- Yes
- No
- Don't know

Were there specific aspects of the checklist and guide that you found helpful?

____

Were there any aspects of the checklist and guide that you thought could be improved?

	What aspects could be improved? (*check all that apply*)			
	Missing information	Unnecessary information	Unclear wording	Other improvement (*please specify*)
Essential elements				
Additional elements				
Explanations				
Checklist as a whole				

Please elaborate on how the checklist and guide could be improved:

____

Do you have any feedback on the examples included in the checklist and guide (see pages 22-29)?

____

Do you have any additional examples to suggest?
(Examples could be of complete or incomplete reporting, relevant to one or more of the checlist items)

____

Would you recommend the checklist to others?

- Yes
- No
- Don't know

Piloting the SynQ checklist and guide

Did you pilot the SynQ checklist and guide?

- Yes
- No

How did you use the checklist?

- To draft text for a protocol
- To draft text for a review
- To check the reporting in a protocol
- To check the reporting in a review
- Other (*please specify*)

Did you check the reporting in:

- Your own protocol or review
- Someone else's protocol or review

Which protocol or review did you use?

- Title:____
- URL (if available):____

Did you find the checklist easy to use?

- Extremely easy
- Somewhat easy
- Neither easy nor difficult
- Somewhat difficult
- Extremely difficult

Do you have any additional comments to add about the content of the SynQ checklist arising from the pilot process? ____

Did you revise or add text to the protocol/review as a result of using the checklist?

- Yes
- No

Did you change your reporting (or suggest a change) based on the checklist?

____

Section B: SynQ (Synthesis Questions) reporting checklist and guide: Instructions for piloting

Thank you for volunteering to provide feedback on the draft SynQ reporting checklist and guide for defining the synthesis questions to be addressed systematic reviews of interventions.

These instructions are for those who wish to pilot the checklist on a review at any stage of development.

To pilot the SynQ checklist and Guide:

1. **Read** the SynQ checklist and guide.
2. **Select** a review you wish to use for the pilot. This may be a review you are authoring yourself, a review you are involved in as an Editor or referee, or another review with which you are familiar. The selected review can be at any stage (i.e. planning, conduct, editorial or published), and can be a Cochrane or other review.
3. **Apply** the SynQ checklist to your selected review, identifying text in the protocol or review that you think meets the requirements of each item, and noting any items not already addressed in the text.
4. Optionally, you may wish to **draft** text that, if added to the protocol or review, would address any checklist items not already met.
5. **Submit** your feedback via the online form.

The process of applying the checklist to a review may take up to two hours in addition to completing the online feedback form, depending on whether you opt to draft additional text.

You will be asked to confirm your consent to participate in this project on the first page of the online feedback form.

Next steps

If possible, we would like to receive the first round of feedback by **Thursday, 14 April 2022**, in order to be incorporated into the next version of the Checklist and Guide. For those who wish to provide feedback after this date, the online form will remain open until Tuesday, 31 May 2022.

A Web Clinic will be hosted by the Cochrane Methods Support Unit on 14 April 2022, where the SynQ checklist and guide will be presented. Register for this event at methods.cochrane.org/about/methods-support-unit/methods-support-unit-web-clinic-schedule.

Where can I get further information?

To discuss the project, please contact Miranda Cumpston (miranda.cumpston@monash.edu).

We thank you for your time and expertise.

Sue Brennan and Joanne McKenzie (principle investigators), Miranda Cumpston (investigator; contact for feedback/piloting), and Rebecca Ryan (investigator)
